# Supplementary material for: Comparison of Wealth-Related Inequality in Tetanus Vaccination Coverage before and during Pregnancy: A Cross-Sectional Analysis of 72 Low- and Middle-Income Countries
Source: Vaccines (Basel). 2024 Apr 17;12(4):431. doi: 10.3390/vaccines12040431 (PMC11054082; doi:10.3390/vaccines12040431)
Supplement: Supplementary file 1 [file vaccines-12-00431-s001.zip › Supplemental Figures S1-S4.pdf]

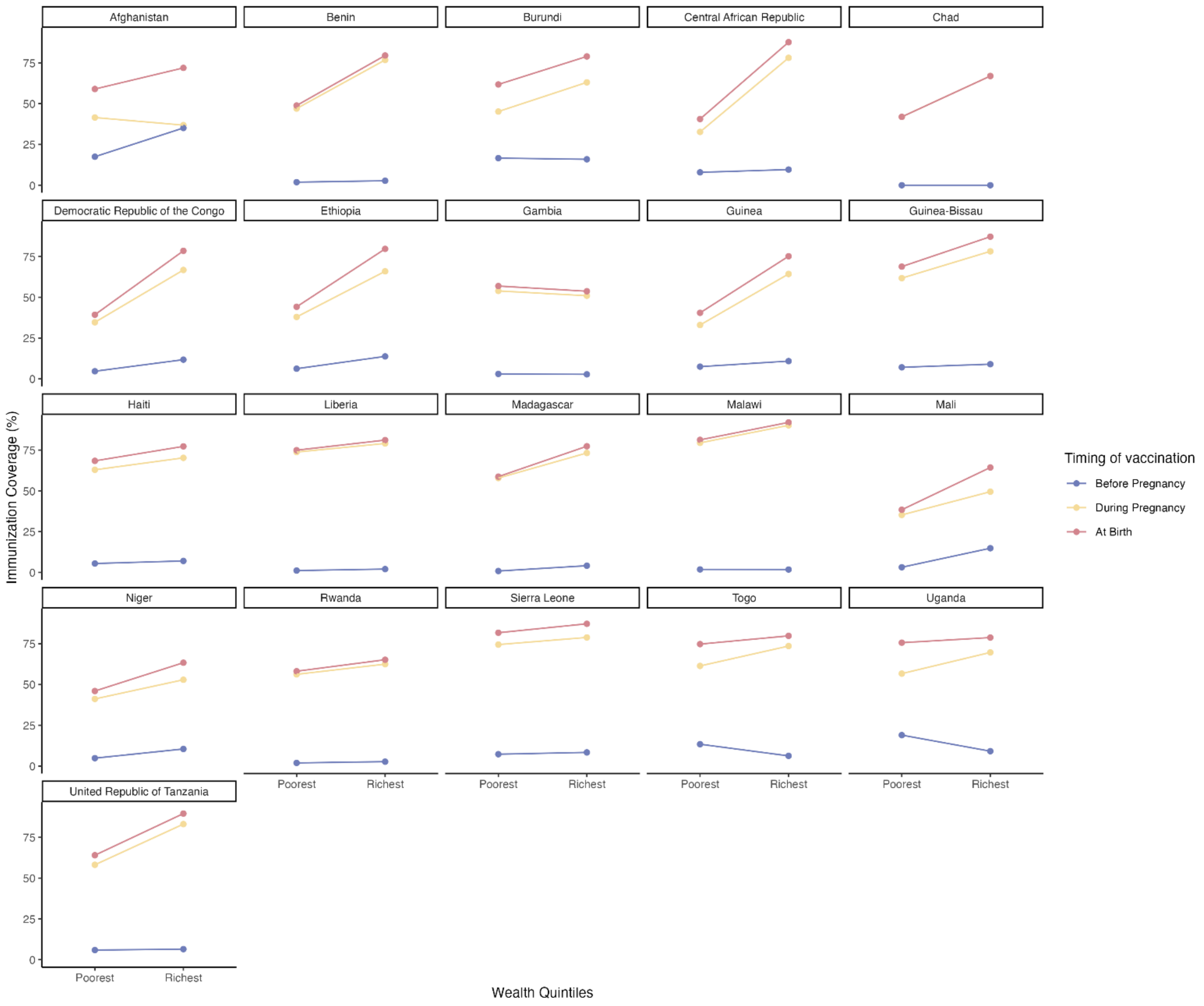

Figure S1: Tetanus immunization coverage before pregnancy, during pregnancy, and at birth, in the wealthiest and least wealthy wealth quintiles in 21 low-income countries

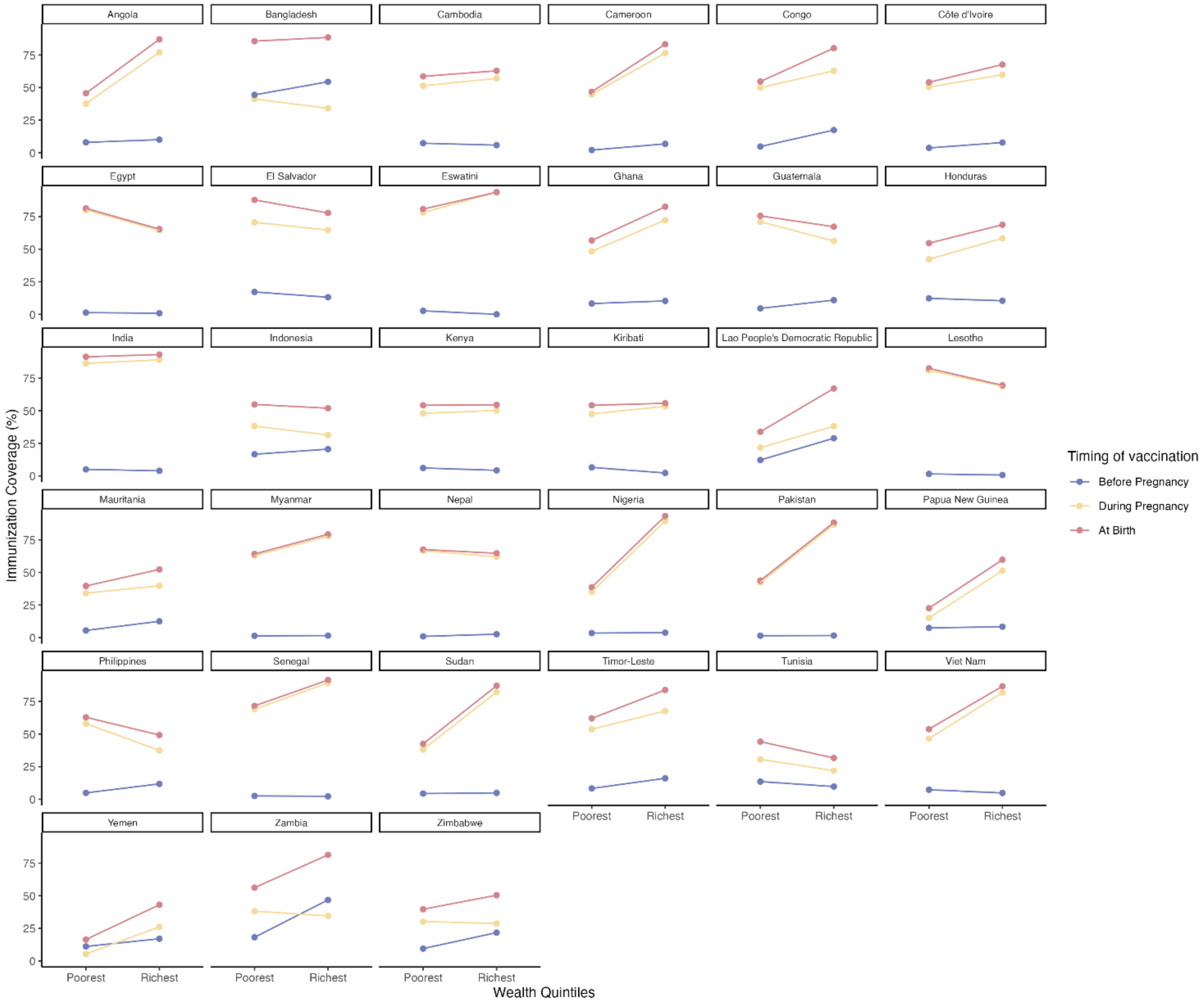

Figure S2: Tetanus immunization coverage before pregnancy, during pregnancy, and at birth, in the wealthiest and least wealthy wealth quintiles in 33 lower-middle income countries

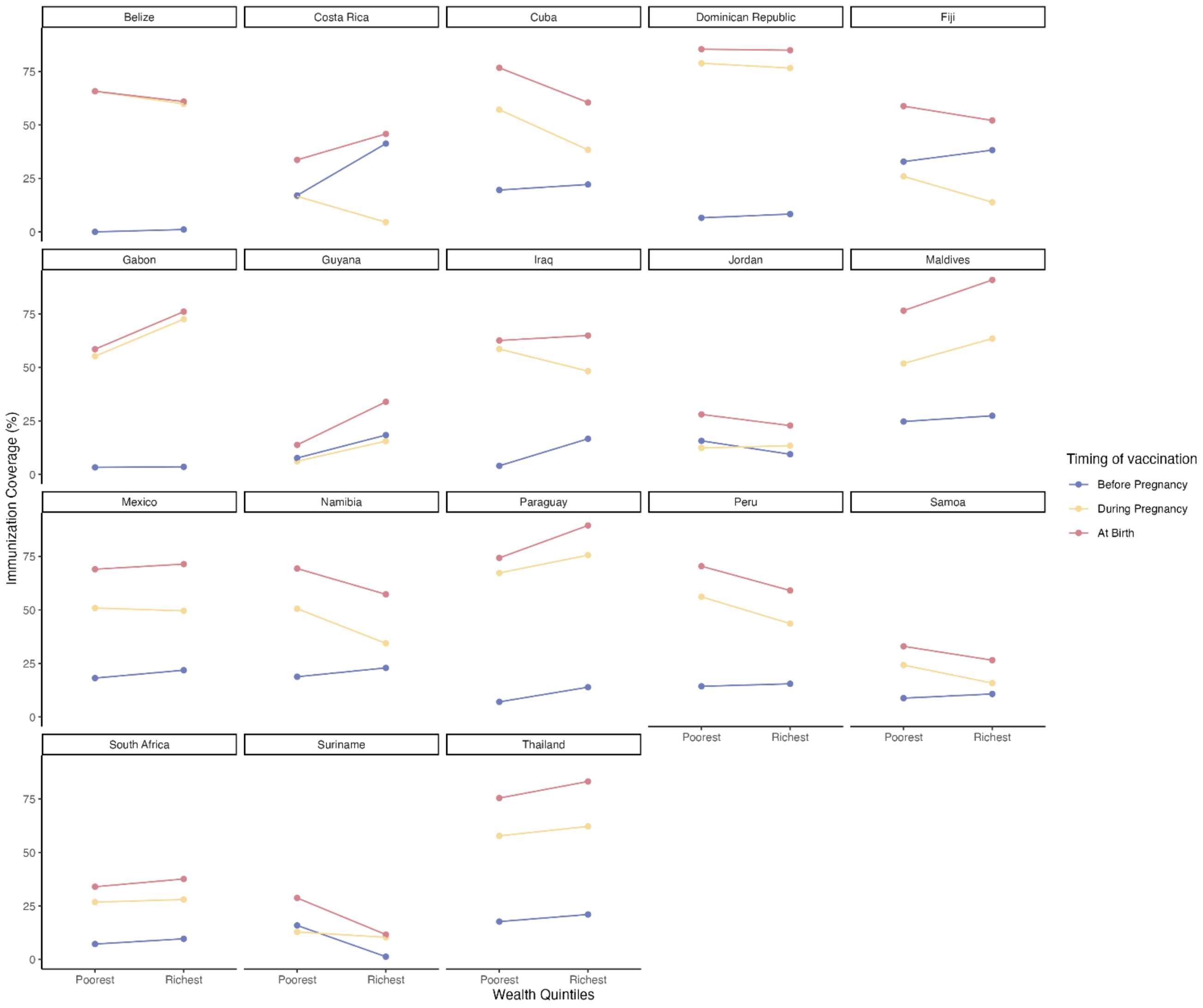

Figure S3: Tetanus immunization coverage before pregnancy, during pregnancy, and at birth, in the wealthiest and least wealthy wealth quintiles in 18 upper-middle income countries

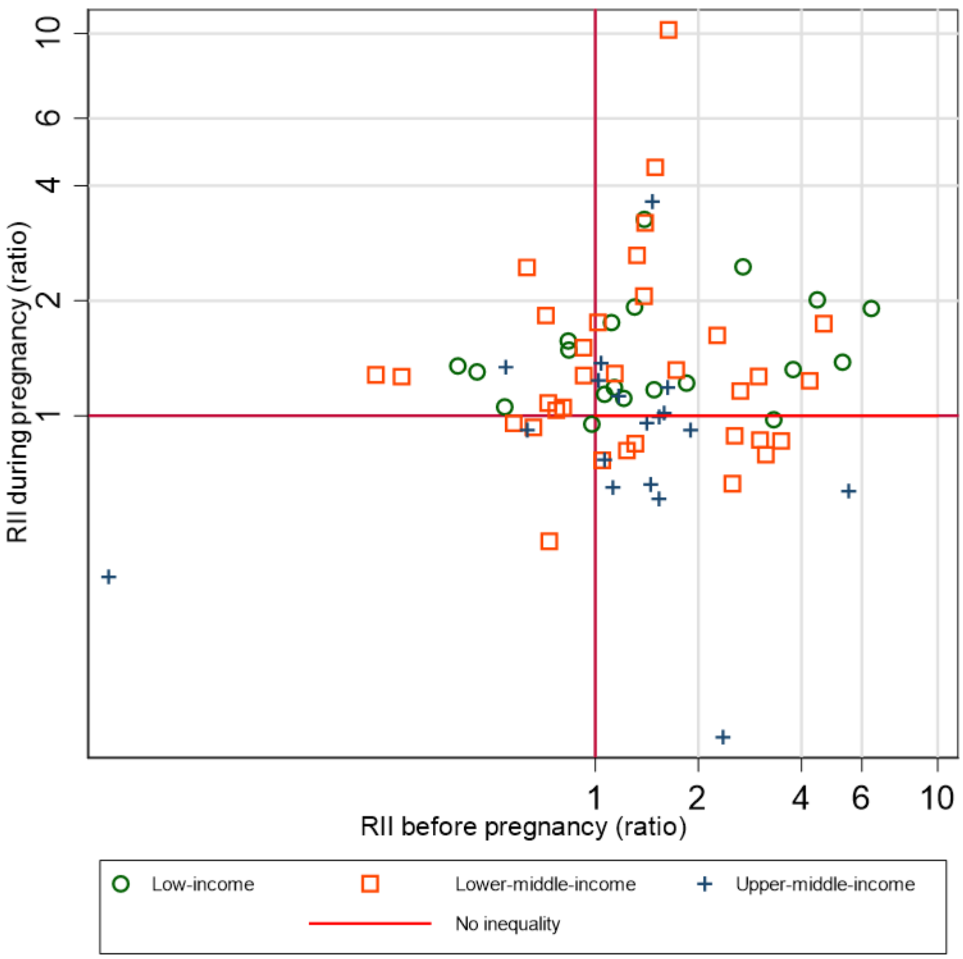

Figure S4. Relative inequality (RII) in maternal tetanus immunization coverage by wealth quintile, before versus during pregnancy
